# Covalent Attachment of Enzymes to Paper Fibers for Paper-Based Analytical Devices

**DOI:** 10.3389/fchem.2018.00214

**Published:** 2018-06-27

**Authors:** Alexander Böhm, Simon Trosien, Olga Avrutina, Harald Kolmar, Markus Biesalski

**Affiliations:** ^1^Laboratory of Macromolecular Chemistry and Paper Chemistry, Department of Chemistry, Ernst-Berl Institute of Chemistry, Technische Universität Darmstadt, Darmstadt, Germany; ^2^Laboratory of Biochemistry, Department of Chemistry, Clemens-Schöpf Institute of Chemistry, Technische Universität Darmstadt, Darmstadt, Germany

**Keywords:** enzyme immobilization, biofunctional paper, lab-on-paper, microfluidics, paper-based diagnostics, point-of-care diagnostics, glucose sensor

## Abstract

Due to its unique material properties, paper offers many practical advantages as a viable platform for sensing devices. In view of paper-based microfluidic biosensing applications, the covalent immobilization of enzymes with preserved functional activity is highly desirable and ultimately challenging. In the present manuscript, we report an efficient approach to achieving the covalent attachment of certain enzymes on paper fibers via a surface-bound network of hydrophilic polymers bearing protein-modifiable sites. This tailor-made macromolecular system consisting of polar, highly swellable copolymers is anchored to the paper exterior upon light-induced crosslinking of engineered benzophenone motifs. On the other hand, this framework contains active esters that can be efficiently modified by the nucleophiles of biomolecules. This strategy allowed the covalent immobilization of glucose oxidase and horseradish peroxidase onto cotton linters without sacrificing their bioactivities and performance upon surface binding. As a proof-of-concept application, a microfluidic chromatic paper-based glucose sensor was developed and achieved successful glucose detection in a simple yet efficient cascade reaction.

## Introduction

Within the last decade, paper and paper-based materials have attracted significant interest as a valuable platform for the development of so-called “lab-on-paper” devices, particularly those focused on paper-based microfluidics (Whitesides, 2006; Yetisen et al., 2013; Xu et al., 2016; Böhm and Biesalski, 2017; Gong and Sinton, 2017). This trend is easily explainable based on the consideration that paper possesses a wide range of interesting material properties. First, it consists mainly of cellulose, which is the most abundant, renewable biopolymer on earth. Additionally, compared to the production of textiles, the preparation of highly porous low-density fiber mats is well-known and highly optimized from a technological point of view. Furthermore, non-woven paper shows almost no thermal expansion, which can be beneficial for further chemical modification of the material through various well-established methods. Thus, paper can be tailored toward defined chemical or physical properties, such as wettability, permeability or reactivity, of the base material (Bracher et al., 2010; Liana et al., 2012). Another highly important benefit of this material for analytical device development is its porous structure, which allows the transport of fluids by capillary forces, making it valuable for microfluidic applications including “low-instrumented” lateral flow tests. Recently, numerous studies that focus on the development of novel paper-based sensors with sensitivity, specificity, and assay diversity have been reported and are being continuously improved (Rolland and Mourey, 2013; Xu et al., 2016; Böhm and Biesalski, 2017; Gong and Sinton, 2017). To date, such paper-based microfluidic sensors have been applied in the first demonstrations in blood typing (Khan et al., 2010; Al-Tamimi et al., 2012; Then et al., 2015) and the analysis of glucose and proteins (Martinez et al., 2007, 2008a,b; Abe et al., 2008; Bruzewicz et al., 2008), urine, (Dungchai et al., 2009; Carvalhal et al., 2010; Yu et al., 2011), pathogenic bacteria (Li et al., 2011; Jokerst et al., 2012), cholesterol (Nie et al., 2010a), and lactate (Dungchai et al., 2009; Nie et al., 2010a; Li and Liu, 2014) as well as in the environmental monitoring of metal ions (Apilux et al., 2010; Nie et al., 2010b; Hossain and Brennan, 2011) and food quality control [e.g., detection of ethanol (Nie et al., 2010a) or pesticides (Hossain et al., 2009a,b; Jahanshahi-Anbuhi et al., 2012)]. In many cases, it is crucial to immobilize a reactive module onto the lignocellulosic fibers. For the development of highly sensitive and specific sensor systems comprising paper-immobilized biomacromolecules, in particular, the nature of the linker connecting the active functionalities, such as antibodies or enzymes, to the surface of the chosen substrate plays a key role, as the activity of the biomolecule can be significantly altered by various interactions with the substrate (Garvey et al., 2017; Huang et al., 2017).

For the immobilization of (bio)molecules on cellulose, three different strategies have been reported to date. In many studies, the molecules are simply physisorbed on the cellulose fibers, thus typically forming weak, non-covalent bonds between the surface and the functional molecule. This method, which is experimentally simple to perform, was further applied toward thermally stable, highly sensitive assays, among others, by printing a pullulan-based ink onto paper surfaces, thus providing the possibility for the stable immobilization of enzymes (Kannan et al., 2015). However, the use of a physisorbed material in microfluidic applications always comes with the limit of possible bleeding of the molecules during the flow test, thus causing low reproducibility and accuracy of the results. In addition, the non-specific adsorption of enzymes within the porous structure of paper can lead to a random orientation and distribution of the molecules, which may lead to reduced accessibility of the active moieties (Garvey et al., 2017). Better control over the orientation of biomolecules can be achieved by using the bioaffinity approach, which does not affect the activity of the immobilized biomolecule (Credou and Berthelot, 2014). However, the required modification of both immobilization counterparts with affinity tags significantly increases the technological complexity in the preparation of such paper devices according to this strategy (Hussack et al., 2010). In a third approach, the linkage of biomolecules to surfaces is achieved by strong, chemically and thermally stable covalent bonds between the biomolecule and the cellulose fibers. This technique requires a chemical reaction between the functional groups on cellulose fibers and the biomolecule.

The covalent immobilization of molecules on lignocellulosic fibers can be accomplished in several ways. One approach relies on the direct functionalization of the hydroxyl groups of cellulose with a biomolecule of choice. If the surface chemistry of the cellulose fibers is not suitable for the straightforward attachment of the desired biomolecular construct, it can be altered, e.g., by transformation of the hydroxyl groups to carboxylic acids (Arola et al., 2012; Orelma et al., 2012b), epoxides (Arola et al., 2012), aldehydes (Credou and Berthelot, 2014; Uth et al., 2014), or more specialized maleimide-decorated cellulose-binding peptides that can undergo light-controlled cycloadditions (Wilke et al., 2017) and active ester groups [*N*-hydroxy-succinimide (NHS)] (Orelma et al., 2012a). Although the successful immobilization of biomolecules has been demonstrated in several examples, there are still a number of challenges ahead of us for deepening our fundamental understanding of the distinct anchoring of biomolecules on paper fibers. For instance, until now, most strategies have relied on chemical modification of the hydroxyl groups of the fibers. However, different papers comprise different fiber sources, and as such, one must take into account the differences in the fiber surface chemistries, including the type of OH groups and their location (in amorphous vs. more crystalline regions). The latter will impact the accessibility as well as the reactivity, and thus, the reproducibility of the modification step is critical. A possible way to overcome some of these limitations is the use of chemically defined precoatings of the fibers, where a chemically stable linkage is provided by non-specific reactions that do not rely on the OH groups of the paper fibers. At the same time, such precoatings should enable the simple and specific binding of a biomolecule of choice.

In recent studies, we used photoreactive copolymers carrying small amounts of photocrosslinkable 4-methacryloyloxybenzophenone (MABP) to functionalize a variety of paper substrates with either hydrophobic, hydrophilic, neutral, or charged surface coatings (Böhm et al., 2013a; Bump et al., 2015) (Böhm, 2014; Wendenburg et al., 2017). The modification process of the fiber surface uses a simple yet very efficient CH-insertion reaction, in which a UV light-excited benzophenone moiety is linked to any aliphatic groups in close vicinity. These aliphatic groups may originate from cellulose, hemicellulose, or lignin polymers, which can be present at the outer surface of the paper fiber. Furthermore, the radical reaction leads to homocrosslinking of the polymer, which results in the formation of a stable polymer network. Proving that covalent bonds are formed between cellulose and the copolymers is not trivial. Methods such as solid-state NMR or DNP (dynamic nuclear polarization) NMR have been used in the past for cellulose characterization, yet there are still limitations with respect to the S/N ratio in the examination of the surface chemistry of paper fibers. Nevertheless, in a number of previous studies, our group has addressed the proof of chemical bonding by indirect measures involving extensive extraction studies using fluorescently labeled polymers and confocal fluorescence microscopy. We were able to show that the non-bound polymers (i.e., non-illuminated, physisorbed) could be easily washed out of the paper sheets in a quantitative manner, whereas the illuminated polymers (i.e., fiber-attached macromolecules) could not be removed. These studies were correlated with the results of confocal Raman microscopy and spectroscopy, showing that the copolymers even photocrosslinked between individual fibers. For details, see Janko et al. (2015) or Bump et al. (2015).

The approach of establishing a functional polymeric coating with photoreactive copolymers offers a large variety of functional copolymers for use in similar fashion for fiber surface modification. Among these copolymers are polymeric architectures decorated with fluorescence markers (Böhm et al., 2013a; Bump et al., 2015), active esters (Böhm, 2014; Wendenburg et al., 2017), or stimuli-responsive copolymers (Li et al., 2016). Because the polymer is coupled by a photochemical strategy, the application of photolithographic masks enables controlled local immobilization of the polymer within defined areas (Böhm et al., 2013b). As was shown in previous studies, the polymer coating homogenously binds to the fiber surface, and the open porous structure of the paper substrate is not significantly altered upon polymer binding (Bump et al., 2015; Janko et al., 2015). The attached macromolecule accumulates at the fiber-fiber contacts during drying of the polymeric solution, thus yielding additional wet-strengthening properties by the cross-linking of individual fibers (Jocher et al., 2015). The latter is an additional benefit for aqueous-based analytical devices. Provided that the copolymers are soluble in water, the fiber-attached polymer networks behave as hydrogels, and their swelling degrees are determined by the cross-linking density. A highly swollen (hydro)gel carrying reactive sites such as active esters is a prerequisite of further functionalization, e.g., with proteins (Park et al., 1995).

With respect to the design of protein-functionalized paper substrates, application of the above-described photocrosslinked polymeric coatings has yet not been reported. In particular, if one considers the possibility of spatially confining such polymers into microfluidic papers, simple cascade reactions involving proteins (enzymes) could potentially be interesting for the design of paper-based analytical devices. In addition to the chemical linkage of the polymer itself, it is of course also interesting to learn about the subsequent protein attachment to the polymer and if the bonding of the biomacromolecule to the hydrogel inside the paper affects or interferes with the desired (bio)function. To address these fundamental questions, we first synthesized monomers carrying active ester functions, and we then copolymerized these monomers with photocrosslinkable comonomers as well as a large amount of the hydrophilic matrix monomer dimethyl acrylamide (DMAA) by free-radical copolymerization. The resulting statistical copolymers were attached to model cotton linters paper by photochemical means. In a second step, the polymer-modified paper was functionalized with enzymes through a transamidation reaction. The influence of the linkage on the enzyme activity was further examined for glucose oxidase (GOx) and peroxidase (POx) in a model reaction consisting of the colorimetric detection of glucose. Finally, as a first proof-of-concept application, we developed a microfluidic glucose sensor that utilizes an enzyme-catalyzed cascade reaction relying on the abovementioned enzymes.

## Materials and methods

### Reagents and methods

All reagents were of high purity and obtained from commercial suppliers (for details, see Supplementary Material). Solvents were distilled prior to use and, if necessary, desiccated by using standard methods. As a model paper substrate, commercially available Roth Rotilabo® 15A filter paper was used (grammage: 84 g m^−2^, BET area: 1.2 g m^−2^, mean pore diameter: 4.6 μm). The synthesis of the monomers used for the preparation of the functional copolymers is described in detail in the Supplementary Material. In a similar fashion, the details of the copolymerization can be found in the Supplementary Material. Furthermore, the preparation of the microfluidic channel and all the characterization methods are described in detail in the Supplementary Material.

### General procedure for the light-induced immobilization of MABP-containing polymers on paper

**Figure 1:**
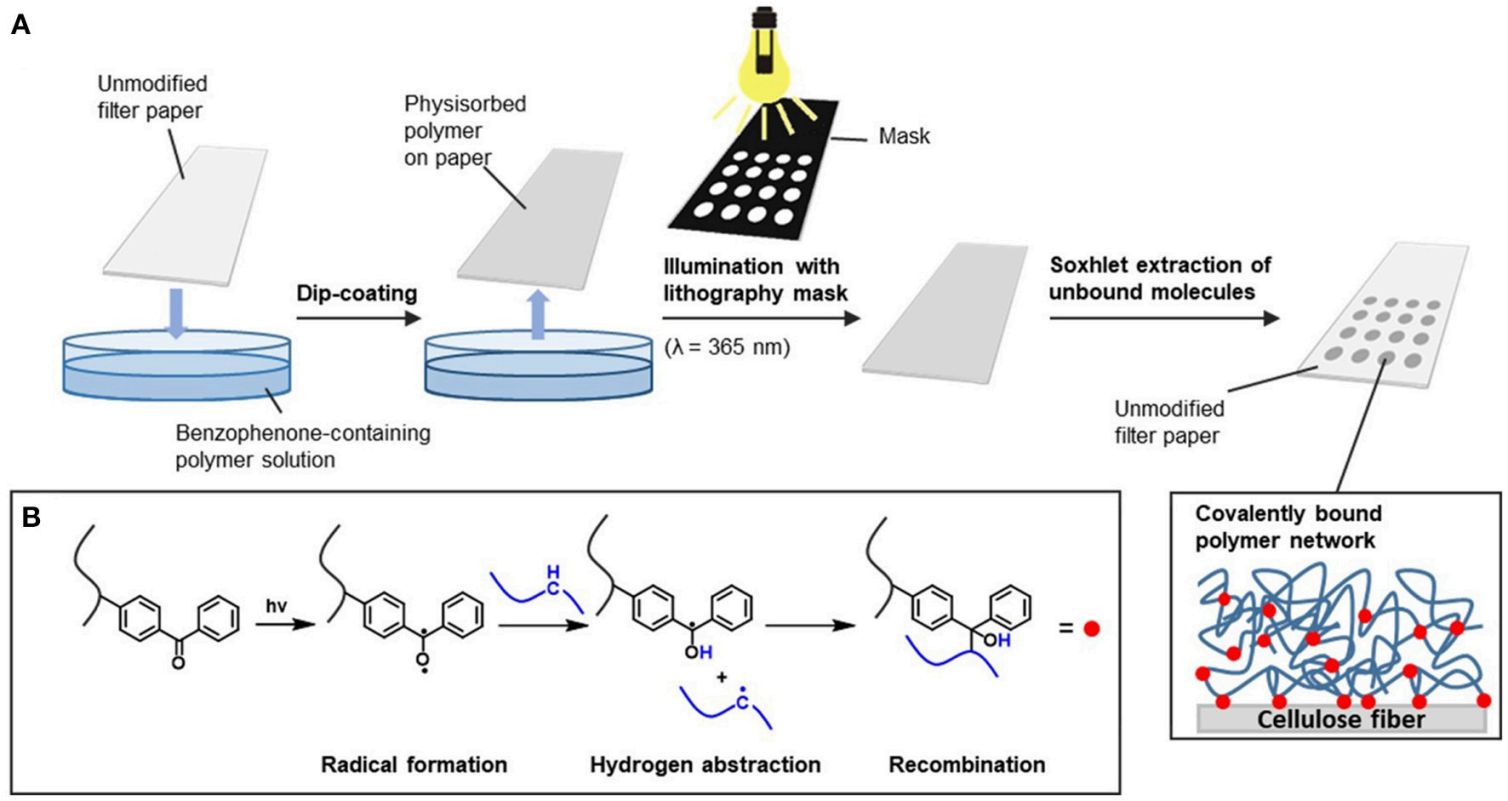
Light-induced immobilization of benzophenone-containing polymers on paper fibers: **(A)** general procedure for photocrosslinking with a lithography mask; **(B)** mechanism of light-induced crosslinking of the benzophenone moieties.

The preparation of polymer-functionalized paper substrates and the photochemical reaction of the benzophenone-functionalized macromolecules are illustrated in Figure 1. In brief, for the covalent attachment of 4-methacryloyloxybenzophenone (MABP)-containing copolymers to the paper fibers, sheets were cut into fragments of the desired size and shape. The DMAA-based copolymers were dissolved in CHCl_3_ at a concentration of 30 mg ml^−1^, whereas the more hydrophobic copolymers, such as the polystyrene-based copolymers, used for the design of the microfluidic barriers were dissolved in THF at the same concentration (30 mg ml^−1^). The paper substrates were dip-coated with the appropriate polymer solution (submerged for 20 s) and air-dried for 30 min. Next, the materials were placed inside a UV exposure chamber and illuminated by UV light (wavelength λ = 365 nm, energy dose E = 16 J cm^−2^) to induce crosslinking of the polymers. To generate well-defined λ = 365 nm, energy dose E = 16 J cm^−2^) to induce crosslinking of the polymers. To generate step (for detailed information, see the Supplementary Material). Finally, unbound polymers were removed by Soxhlet extraction with CHCl_3_ or THF for 2.5 h. Before further use, all samples were air-dried and stored in a climate room (50% rel. humidity and 23°C) for at least 24 h.

## Results and discussion

### Spatially resolved functionalization of paper substrates with model amines by using active ester-containing photocrosslinkable polymers

To chemically attach the photoreactive active ester-containing copolymers (P(DMAA-*co*-MAC_2_AE-*co*-MABP)) to the paper fibers, small pieces of filter paper (2.5 × 2.5 cm) were first submerged in a solution of the functional polymer in CHCl_3_ (30 mg ml^−1^) for 20 s (dip-coating). After the coated substrates were dried, the samples were illuminated by UV light using an Oriel 1,000 W flood exposure chamber (λ = 365 nm) with E = 16 J cm^−2^, at which 94% of the present benzophenone groups were excited (double-sided illumination) (Berchtold, 2005). After illumination, the unbound macromolecules were removed by Soxhlet extraction for 2.5 h with chloroform. It has been shown in previous studies that after this extraction, no physisorbed polymer material remains inside the substrates (Böhm et al., 2013b).

**Figure 2:**
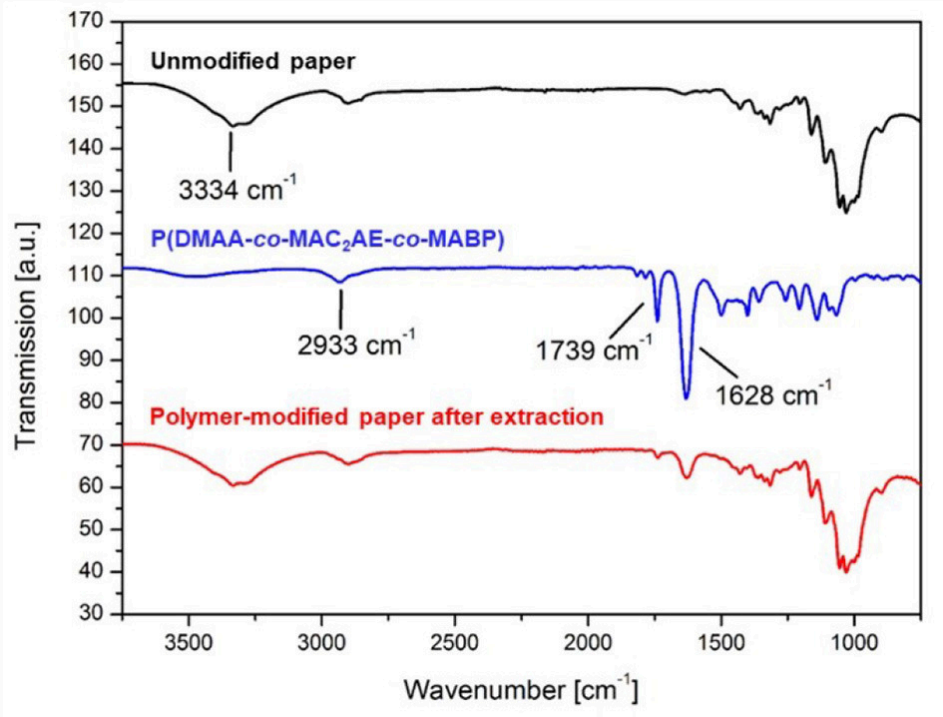
Comparison of the IR spectra of unmodified filter paper (top, black line), P(DMAA-*co*-MAC_2_AE-*co*-MABP) bulk polymer (middle, blue line) and P(DMAA-*co*-MAC_2_AE-*co*-MABP)-modified filter paper (bottom, red line).

To analyze the polymer attachment to the paper fibers with respect to the chemical identity, the functionalized substrates were examined by ATR-FTIR spectroscopy (see Figure 2). Comparison of the IR spectra of the unmodified filter paper (top, black line), the P(DMAA-*co*-MAC_2_AE-*co*-MABP) bulk polymer (middle, blue line) and the corresponding polymer-modified filter paper (bottom, red line) was conducted. The absorption band at 3,334 cm^−1^ is attributed to the stretching vibration of the OH groups of cellulose. Furthermore, the absorption bands at 2,933, 1,739, and 1,628 cm^−1^ are assigned to the stretching vibrations of aliphatic CH_2/3_ groups (cellulose and polymer), R_2_-CO groups (carbonyl groups in the active ester moieties and benzophenone) and R'-CO-NHR'/R”-CO-NR'_2_ groups [carbonyl groups in the active ester moieties (secondary amide and tertiary amide) and carbonyl groups in the DMAA units (tertiary amide)], respectively. Note that additional characterization of PDMAA-functionalized paper has been recently published elsewhere (Janko et al., 2015; Jocher et al., 2015; Wendenburg et al., 2017), and the reader is referred to those articles for more details.

The mass of polymer attached to the fibers was determined by gravimetric analysis. To obtain quantitative measurements and to reduce possible sources for experimental error, the materials were equilibrated at 23°C and 50% humidity for at least 24 h in a climate room before and after each modification step. The latter ensures the presence of a similar amount of adsorbed water in the samples. Gravimetrical analysis indicated that ~40 mg of polymer per gram of cellulose fibers was attached to the filter paper.

**Figure 3:**
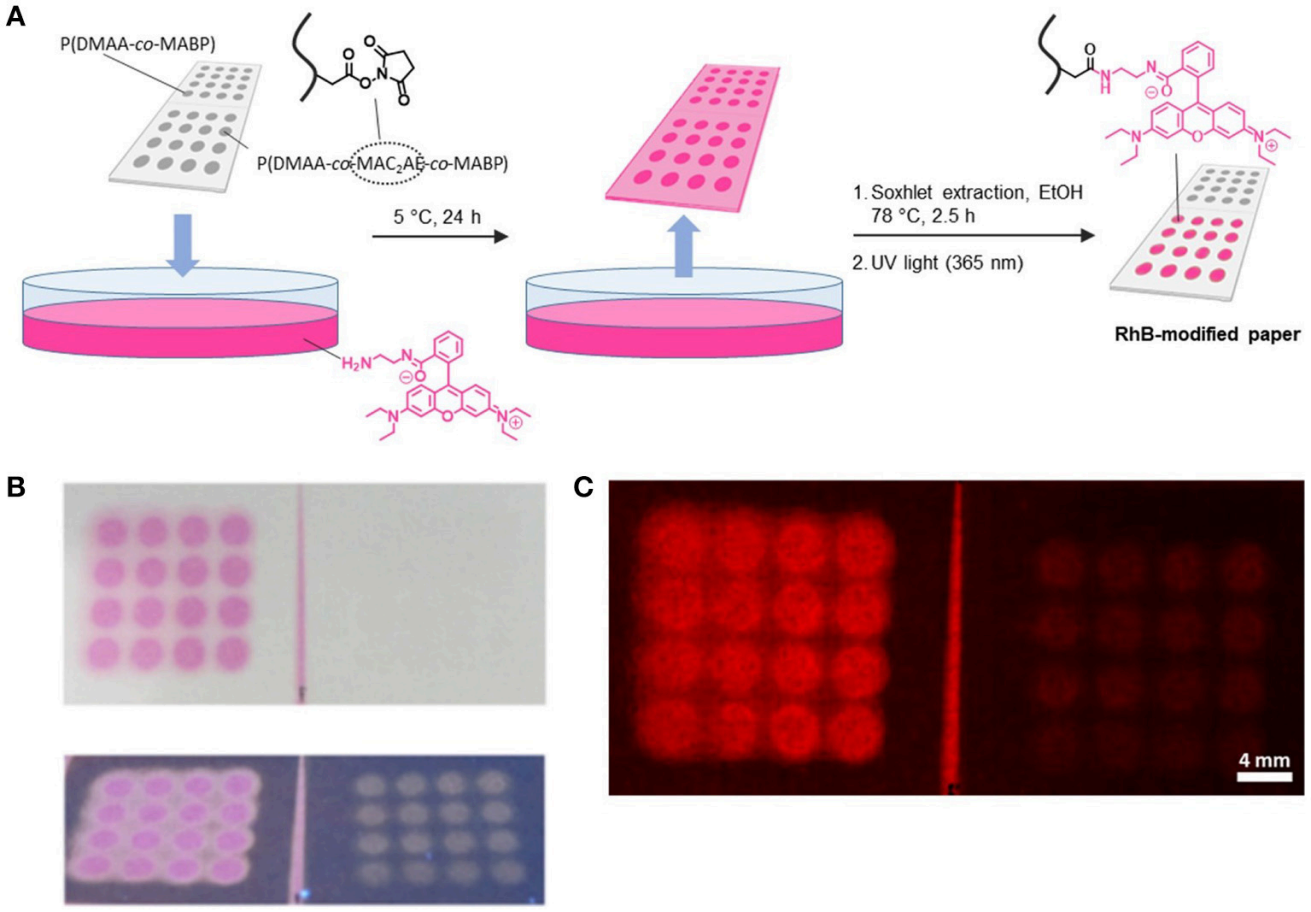
**(A)** Production procedure of RhB-NH_2_-modified polymer patterns on paper by transamidation of active ester moieties with RhB-NH_2_; **(B)** photographs (under visible light and under UV light) and **(C)** fluorescence micrograph of the RhB-modified paper.

Next, we were interested in examining the accessibility of the active ester groups of the polymer-modified filter paper. As a reference model, we first investigated the reaction between the active ester functionalities of the fiber-attached polymer network with a model dye, an amine-bearing fluorescent rhodamine B-based compound (RhB-NH_2_). This model reaction is illustrated in Figure 3A. To investigate the spatially resolved immobilization of the fluorescent dye, model filter paper (5 × 2.5 cm) was first modified with P(DMAA-*co*-MAC_2_AE-*co*-MABP) as described above. Then, the samples were covered with a lithography mask comprising spots 3 mm in diameter during illumination by UV light. In addition, one-half of the paper substrate was completely covered during this illumination period as a reference test area. Hence, after a Soxhlet extraction of the unbound polymer, only half of the paper substrate (2.5 × 2.5 cm) was modified with the functional copolymer. In the next step, the same paper substrate was subjected to a similar modification procedure using the corresponding reference copolymer P(DMAA-*co*-MABP), which contains no active ester functionalities and is therefore not capable of binding an amine in similar specific fashion. After the paper was modified with both types of polymers, the paper substrate was submerged in a solution of the model amine RhB-NH_2_ (2 mg ml^−1^) in ethanol for 24 h at 5°C. After this time, the unbound dye molecules were removed from the paper substrate by Soxhlet extraction with ethanol (78°C, 2.5 h). During the extraction step, a color change of both the extracted and paper-attached dye from pink to orange was observed due the fact that the utilized rhodamine B derivative is thermoresponsive, i.e., the open form of RhB-NH_2_ (fluorescent, pink) is thermally transformed into the closed one (non-fluorescent, pale orange), as previously described by Schäfer et al. (2013). Further illumination of the samples with UV light (λ = 365 nm) for 10 min led to a color change of the immobilized dye from orange to pink due to the conversion of the rhodamine B derivative to its fluorescent open form (photoresponsiveness), and thus, the functionalized polymer spots became visible by the naked eye. Subsequent heating of the sample followed by repeated illumination with UV light led to a color change of the dye from pink to orange and back to pink, indicating the reversibility of the photoactivation process (see Supplementary Material), making this rhodamine derivate an interesting light- and temperature-responsive switch (Schäfer et al., 2013).

Samples were first investigated visually by taking photographs and comparing the images obtained during UV illumination (λ = 365 nm) (Figure 3B). As can be inferred from the images, the areas that contained active ester moieties as a result of modification with the P(DMAA-*co*-MAC_2_AE-*co*-MABP) copolymer, referred to as “NHS Polymer” exhibited strong fluorescence after attachment of the model amine RhB-NH_2_, whereas only very pale fluorescence could be detected in the spots where the reference copolymer P(DMAA-*co*-MABP) was attached (for fluorescence micrographs please see Figure 3C). This pale fluorescence that could be seen within the polymer domains of the reference copolymer in the photographs captured under UV illumination and that was visible even without any treatment with RhB-NH_2_ may be caused by the intrinsic fluorescence of the benzophenone groups present in the copolymer. This finding suggests that the active ester-containing polymer that is covalently bound to the fibers can be used to attach amine-functionalized molecules in a specific fashion.

### Enzyme immobilization and activity studies

After investigation of the attachment of the rhodamine model dye via transamidation, we were next interested in examining the possibility of immobilizing enzymes on the amine-reactive polymer-modified paper substrates. To that end, our model filter paper was decorated with P(DMAA-*co*-MAC_2_AE-*co*-MABP) as described above and submerged in a solution of glucose oxidase (GOx) in phosphate buffer (0.5 mg ml^−1^) for 24 h at 5°C. After removal of the unbound enzymes by thorough rinsing with Millipore water and an aqueous sodium chloride solution (3 M) for 6 h at 5°C, the desired enzyme-modified paper substrate (GOx-modified paper) was further investigated.

**Figure 4:**
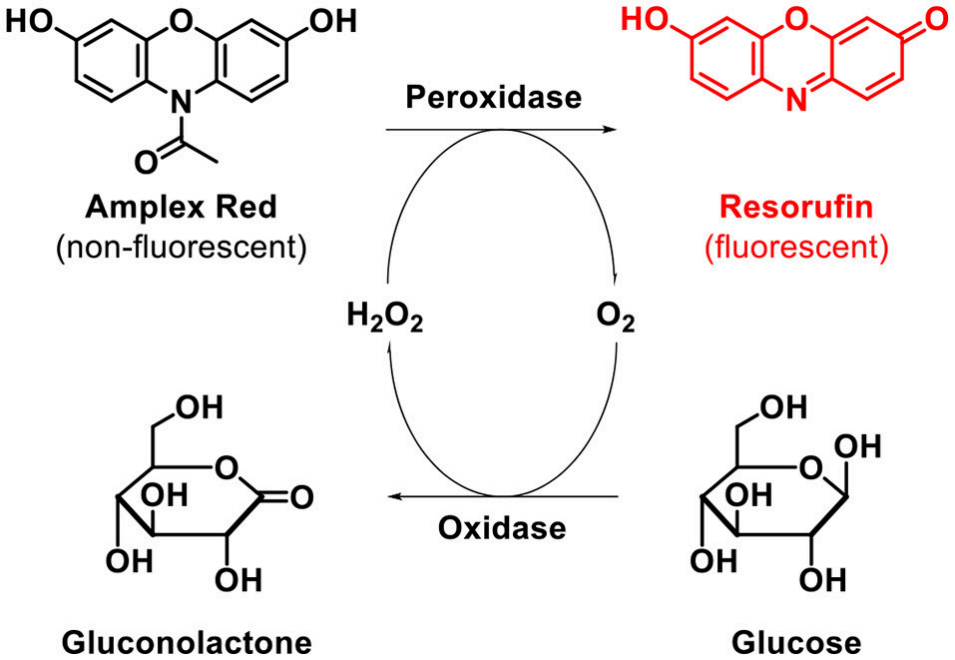
GOx-mediated detection of β-d-Glucose.

We next investigated the activity of the GOx-modified paper in a model cascade assay used for the determination of the β-d-glucose concentration. The cascade reaction involved two steps, as illustrated in Figure 4. In brief, the first step is based on the GOx-catalyzed oxidation of β-d-glucose to d-glucono-δ-lactone. In the second step, the non-fluorescent dye Amplex Red is transformed to its fluorescent counterpart resorufin via a horseradish peroxidase (HRP)-catalyzed reaction with hydrogen peroxide (Figure 4, upper part).

**Figure 5:**
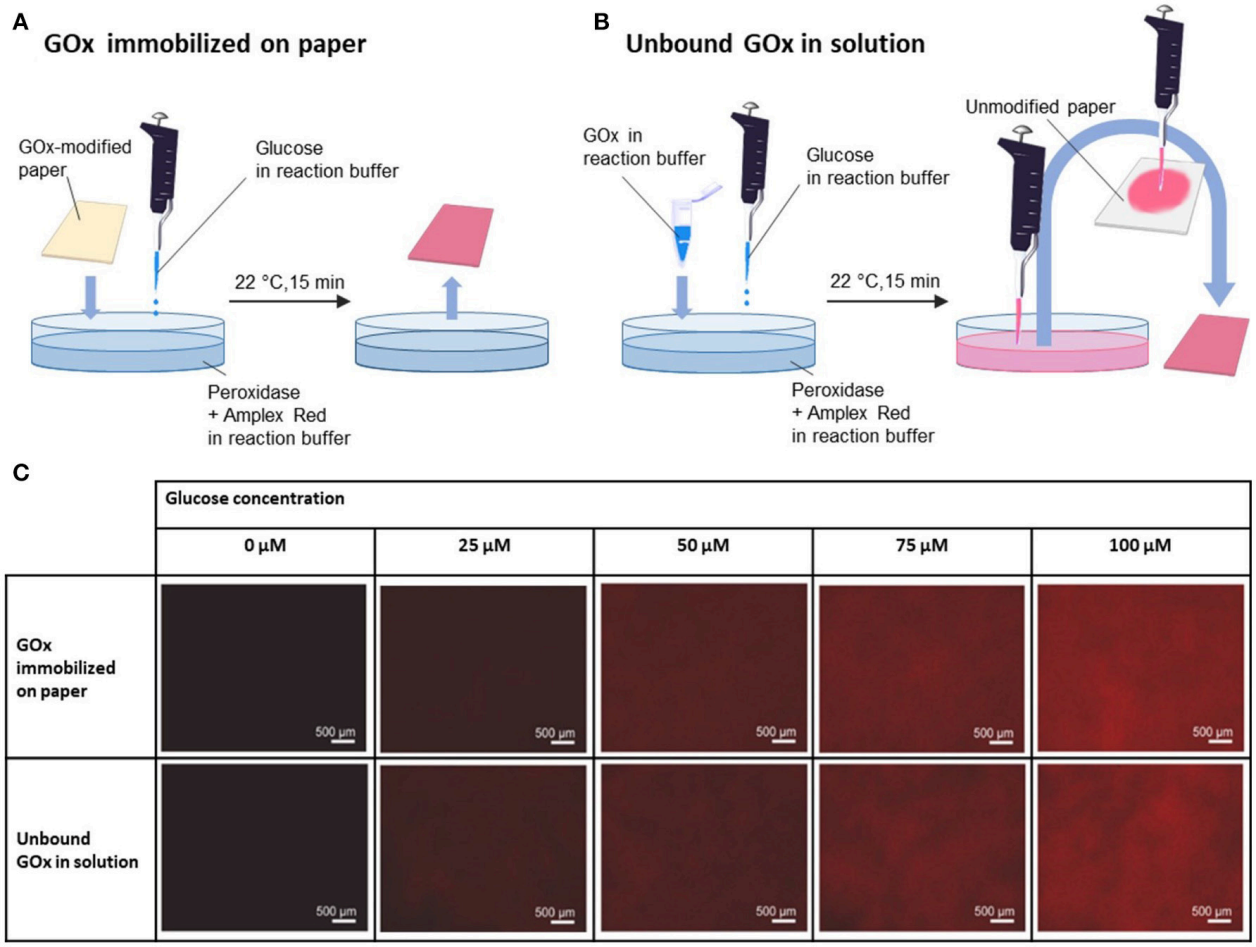
**(A)** Procedure of treatment of GOx-modified paper with glucose; **(B)** procedure of treatment of GOx and glucose in solution and subsequent immobilization on paper as a reference experiment; **(C)** fluorescence micrographs of GOx-modified paper treated with different concentrations of glucose (0, 25, 50, 75, and 100 μM) obtained by the described different production methods.

To adapt this assay to paper, we prepared a series of GOx-modified paper substrates that we then submerged in a buffer solution containing Amplex Red and HRP (see Figure 5A for illustration). We then added glucose at different concentrations of 25, 50, 75, and 100 μM. After 15 min of incubation, the paper was removed from the assay, and the wet weight was determined (without rinsing off the physisorbed resorufin). After drying in air, fluorescence micrographs were captured and are shown in Figure 5C. Note that because resorufin exhibits strong fluorescence, the reaction can be easily monitored even on paper by means of fluorescence microscopy. For reference (Figure 5B), the same experiment was conducted in solution with similar amounts of unbound glucose oxidase (compared to the amount of paper-attached GOx, assuming that all present active ester moieties inside the polymer-modified paper substrates reacted with GOx) and glucose (with the same concentrations as used in the abovementioned experiment). After the solution was incubated again for 15 min, a defined amount of the resulting solution was drop-casted onto unmodified filter paper, and the weight in the wet state was determined. Note that GOx solution-impregnated paper with the same mass as the wet substrate of the GOx-modified paper was prepared by adjusting the applied volume of the assay solution. This procedure enabled simple comparison of the amount of resorufin dye in both cases because in both cases, the dye was only physisorbed to the paper fibers. After drying in air, again, fluorescence micrographs were captured of the impregnated paper (see Figure 5C). As shown in Figure 5C, the obtained fluorescence micrographs for both experiments show similar increases in the fluorescence intensities with an increasing glucose concentration in solution. This result is an indication that the enzyme activity of glucose oxidase is not altered upon immobilization.

**Figure 6:**
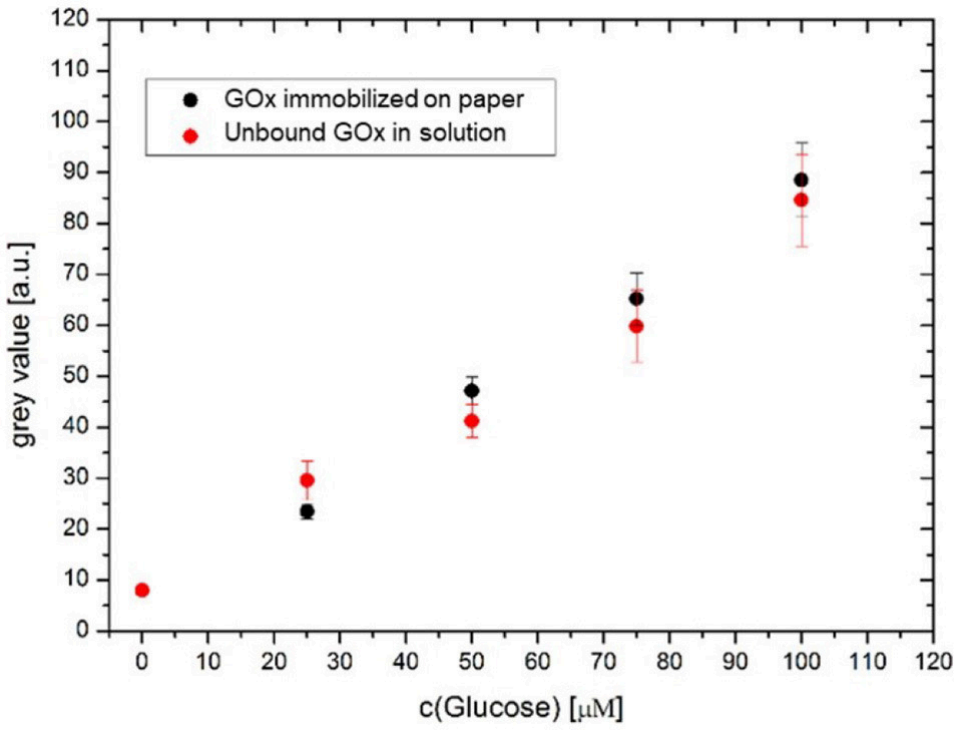
Plot of the gray values of the fluorescence micrographs vs. glucose concentration for the GOx-modified paper (black) and glucose oxidase in solution (red) (the solution was afterwards dropped onto unmodified paper substrates).

To analyze this finding in more detail, the fluorescence intensity of the captured micrographs was further analyzed according to the gray values of the captured images. The measured gray values are shown in Figure 6 as a function of the glucose concentration. As seen from Figure 6, similar gray values were obtained for both experiments, indicating that the enzyme activity was not altered upon immobilization of the enzyme on the surface-attached polymer network of the paper substrates.

### Proof-of-concept studies: enzyme cascade reactions on paper

In the final step, to address the possible engineering of such a simple colorimetric glucose sensor, we designed a microfluidic paper device capable of performing the abovementioned glucose detection assay. To that end, we constructed a microfluidic channel according to our previously described method (Böhm et al., 2013a), making use of photocrosslinkable, hydrophobic, polystyrene-based polymers (P(S-*co*-MABP-*co*-PyMA)), and appropriate lithography methods (channel width of 0.4 cm, see the Supplementary Material for preparation details).

To visualize the channel under UV light illumination, a blue fluorescent pyrene-based monomer (PyMA) was copolymerized with styrene and MABP monomer. After the channel was fabricated, two active ester-containing polymer patches [consisting of P(DMAA-*co*-MAC_2_AE-*co*-MABP-*co*-PyMA)] were fabricated within the channel by using an appropriate photomask (each polymer patch had a length of 1 cm) (see Supplementary Material). Again, PyMA was chosen as a fluorescent marker to visualize the polymer patches under UV light illumination.

**Figure 7:**
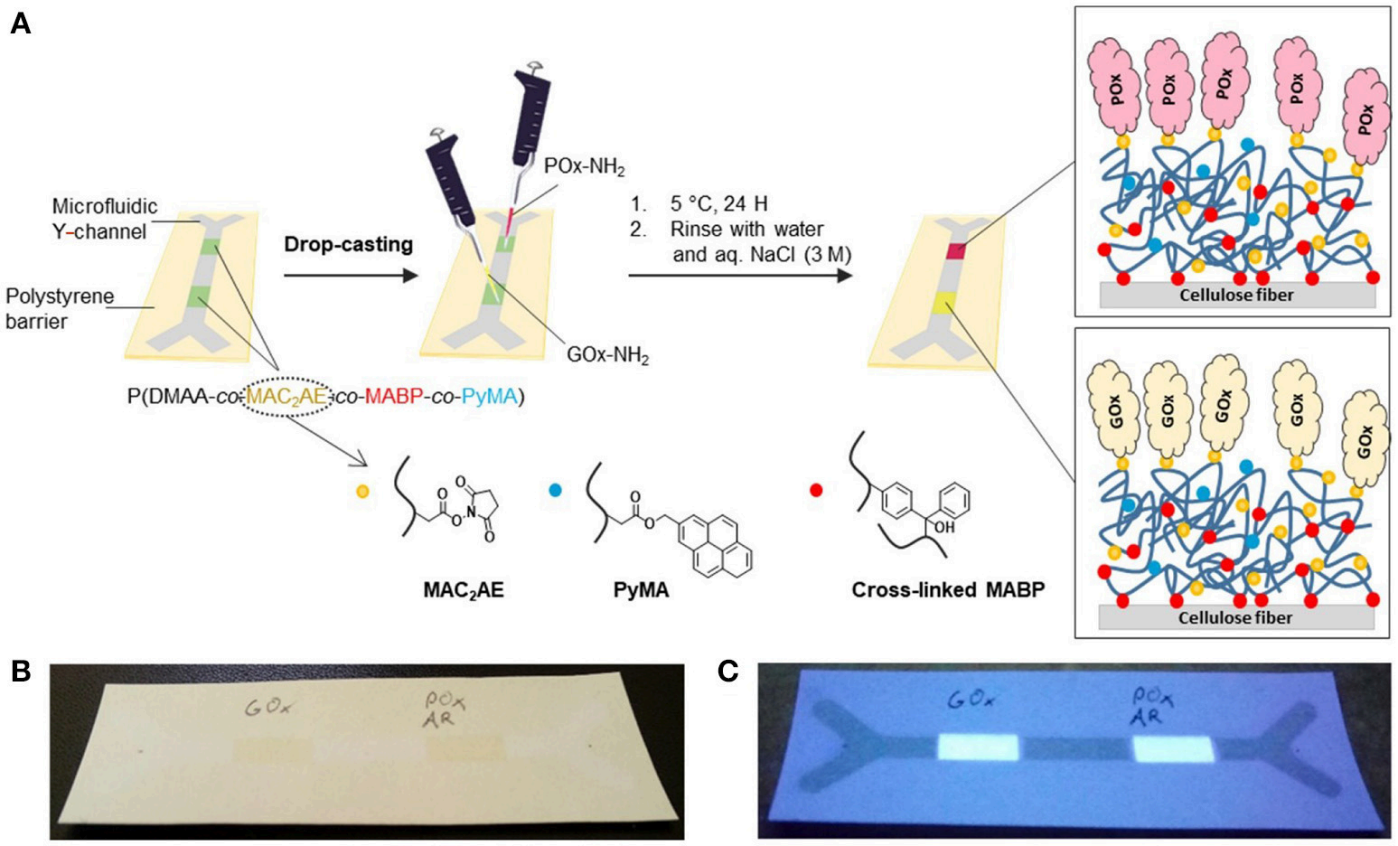
**(A)** Schematic illustration of the functionalization of the active ester-modified paper (“NHS paper”) with GOx-NH_2_ and POx-NH_2_ in well-defined areas (polymer patches) within a microfluidic channel; photographs of the microfluidic Y channel containing GOx- and POx-modified polymer patches after the addition of Amplex Red (AR) under visible light **(B)** and under UV light (365 nm) **(C)**. Before glucose treatment, the POx patch containing AR is colorless and does not show any red fluorescence.

In the next step, both enzymes (GOx and HRP) were covalently attached to the polymer patches inside the microfluidic channel. For this procedure, solutions of the enzymes in sodium phosphate buffer (pH 7.4) were prepared and drop-casted onto the patches. In the case of the glucose oxidase, a solution of 200 units ml^−1^ was prepared in incubation buffer [1 unit of glucose oxidase oxidizes 1 μmol of β-d-glucose to d-glucono-δ-lactone and H_2_O_2_ per minute at pH 5.1 and 30°C^1^]. Additionally, a stock solution of horseradish peroxidase in incubation buffer was prepared [10 units ml^−1^; 1 unit of horseradish peroxidase typically generates 1 mg of purpurogallin from pyrogallol in 20 s at pH 6.0 at 20°C^1^]. To immobilize the enzymes within the polymer-modified channel, 4 μl (~0.8 units) of the GOx solution was drop-casted on top of one polymer patch. Subsequently, 4 μl (~0.04 units) of the HRP stock solution was drop-casted on top of the second polymer patch. The channels were covered to minimize evaporation of the incubation buffer and kept at 5°C for 24 h to covalently attach the enzymes to the paper-attached, photocrosslinked, active ester-containing polymers via transamidation of the activated carboxy groups with the primary amines in the side chains of the enzyme's lysines. Then, the channels were rinsed with multiple portions of water (Millipore) followed by treatment of the channels with an aqueous 3 M NaCl solution for 6 h at ambient temperature to remove the unbound proteins. Finally, the channels were again rinsed with Millipore water to remove the remaining salts prior to the test assay (see Figure 7). In the final step, a stock solution of Amplex Red in anhydrous DMSO (concentration = 0.01 mol l^−1^) was spotted on the HRP-containing polymer patch inside the channel (V = 2.5 μl, m_AR_ = 6.5 μg) to generate the abovementioned indicator system. After channel preparation, Amplex Red did not show any red florescence (see Figure 7C).

To perform the microfluidic assay, the channel was fixed between two PMMA slides, and the two inlets of the Y channel were connected to syringe pumps via silicon tubes. The syringe pumps were used to transport the aqueous β-d-glucose solution (100 μM) through the silicon tubes to the channel inlets. The pressure of the syringe pumps was set to zero once the liquid front had reached the channel inlets, and the analyte-containing solution was transported inside the paper-based channel via capillary forces. Because fluid transport in the filter paper was comparatively slow, the reaction was incubated at room temperature for 60 min to ensure that a sufficiently large amount of hydrogen peroxide produced as a side product during the GOx-catalyzed oxidation of β-d-glucose was transported to the HRP-containing polymer patch. There, the red fluorescent dye resorufin was formed through the HRP-catalyzed oxidation of Amplex Red in the presence of hydrogen peroxide (the channel was kept in the dark during incubation, as Amplex Red is very sensitive to light).

**Figure 8:**
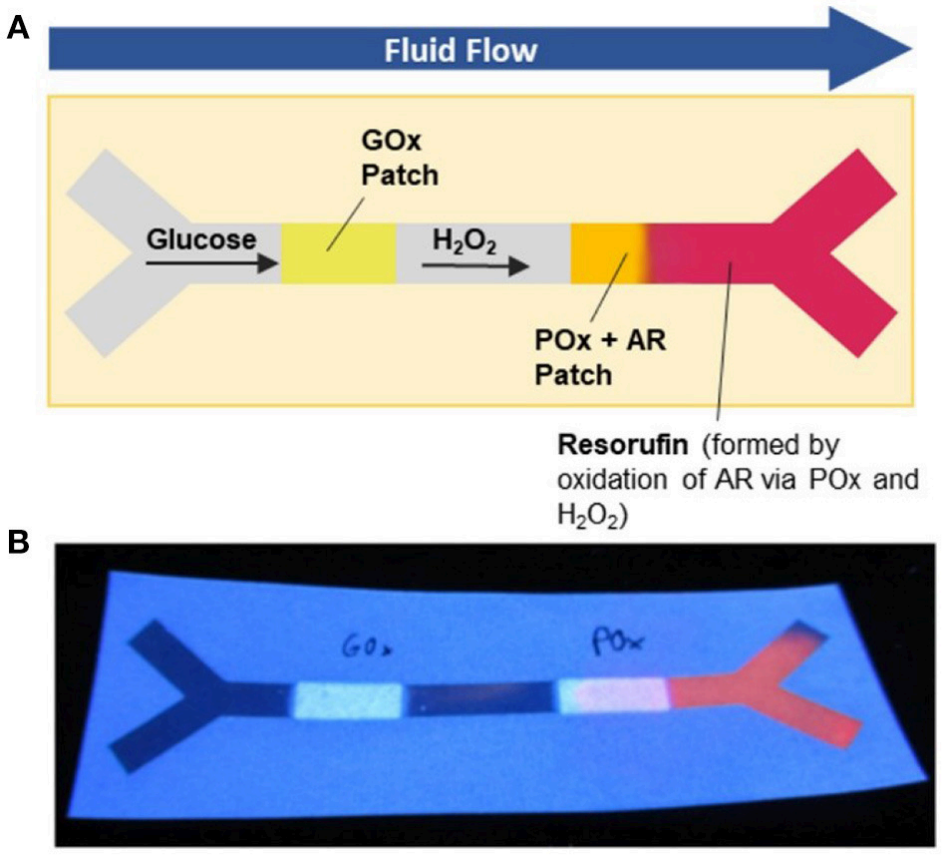
Microfluidic paper-based glucose sensor as a proof-of-concept application: **(A)** Schematic illustration of the work principle: A solution of glucose is transported through the microfluidic channel by capillary forces. The glucose passes the GOx patch, where it is oxidized to a gluconolactone concomitant with the formation of H_2_O_2_. The latter species oxidizes the non-fluorescent Amplex Red dye present within the POx patch to red-fluorescent resorufin; **(B)** photograph of the microfluidic sensor after use.

In this first proof-of-concept study, the reaction was terminated after an incubation time of 60 min (i.e., the channel was disconnected from the inlet tubes), and the channels were air-dried in the dark. After drying, the channels were placed under a UV hand lamp and analyzed via illumination with 365 nm light (Figure 8). As seen from Figure 8, red fluorescence was detected inside the HRP-modified polymer patch as well as inside the two outlets of the channel, indicating the successful formation of the reaction product resorufin after the cascade reaction of β-d-glucose at the GOx patch and H_2_O_2_ at the HRP patch. This observation qualitatively demonstrates the successful immobilization of the two enzymes inside the paper-based channel as well as the activity of the immobilized biomolecules. Such issues as the influence of the immobilization time, washing stability, enzyme concentration during illumination, kinetics of the reaction, detection limits, and influence of the paper parameters on the activity of the enzymes are currently under thorough investigation.

## Conclusions

In conclusion, we demonstrated a novel strategy for the covalent immobilization of proteins (enzymes) onto paper fibers. For this process, hydrophilic, benzophenone-containing copolymers containing active ester functionalities were photocrosslinked onto paper fibers. Subsequently, enzymes were covalently bound to the surface by transamidation. This method enabled the immobilization of a model enzyme, glucose oxidase, onto the cellulose fibers without compromising its activity. In particular, with the chosen glucose assay, the on-paper activities were similar to the off-paper activities, making this assay an interesting candidate for colorimetric glucose sensing. In a final proof-of-concept study, we demonstrated that this method can be easily expanded to the fabrication of microfluidic cascade sensing assays. Thus, we were able to monitor the glucose content using a chromatic paper sensor based on the cascade reaction of the immobilized functional proteins glucose oxidase and glucose peroxidase combined with a fluorescent indicator Amplex Red.

## Author contributions

All authors listed have made substantial, direct and intellectual contributions to the work: AB performed all experiments. AB and ST analyzed the data, reviewed the literature and wrote the manuscript. OA and HK coordinated the enzyme part of the project. MB coordinated the complete project. All authors discussed, reviewed and approved the manuscript.

### Conflict of interest statement

The authors declare that the research was conducted in the absence of any commercial or financial relationships that could be construed as a potential conflict of interest.

## Footnotes

^1^Invitrogen website. *Amplex Red Glucose/Glucose Oxidase Assay Kit*. Available online at: http://tools.thermofisher.com/content/sfs/manuals/mp22189.pdf (Accessed January 25, 2018)
