# Supplementary material for: Covalent Attachment of Enzymes to Paper Fibers for Paper-Based Analytical Devices
Source: Front Chem. 2018 Jun 27;6:214. doi: 10.3389/fchem.2018.00214 (PMC6030327; doi:10.3389/fchem.2018.00214)
Supplement: Supplementary file 1 [file Data_Sheet_1.PDF]

Supplementary Material

Covalent Attachment of Enzymes to Paper Fibers for Paper-Based Analytical Devices

Alexander Böhm, Simon Trosien, Olga Avrutina, Harald Kolmar, Markus Biesalski*

***Correspondence:** Markus Biesalski, biesalski@tu-darmstadt.de

Table of Content

1 Materials and instruments 2

1.1 Materials 2

1.2 Instruments and characterization methods 2

2 Synthesis and production methods 4

2.1 Synthesis and characterization of compounds 4

2.2 Synthesis of polymers 5

2.3 Covalent immobilization of polymers onto paper 7

2.4 Preparation of the glucose sensor 8

3 Reversible switching of RhB-modified paper 9

4 References 9

1 Materials and instruments

1.1 Materials

All reagents for synthesis were used of high purity. Grades and suppliers are listed in the table below:

Reagent	Purity/Grade	Supplier
Amplex Red (AR)		Invitrogen
β-Alanine	98%	Alfa Aesar
2,2′-Azobis(2-methylpropionitrile) (AIBN)	>98%	Fluka
N,*N*-Dicyclohexylcarbodiimide (DCC)	99%	VWR
N,*N*-Dimethylacrylamide	99%	Sigma Aldrich
Ethylene diamine	99%	abcr
β-D-Glucose		Invitrogen
Glucose oxidase (GOx)		Invitrogen
Horseradish peroxidase (HRP/POx)		Invitrogen
4-Hydroxybenzophenone	98%	Alfa Aesar
N-Hydroxysuccinimide	98%	Sigma Aldrich
Methacryloyl chloride	97%	Sigma Aldrich
1-Pyrenemethyl methacrylate	99%	Sigma Aldrich
Rhodamine B base	99%	Acros
Sodium phosphate buffer (pH 7.4)		Invitrogen
Styrene	99%	Acros

N,*N*-Dimethylacrylamide, methacryloyl chloride and styrene were filtered through basic alumina, distilled under reduced pressure and stored under nitrogen atmosphere prior to use. Solvents were used in analytical grades and dried, if necessary, by using standard methods.

As model paper substrate commercially available Roth Rotilabo® 15A filter paper was used (grammage: 84 g m^{-2}, BET area: 1.2 m^{-2}, mean pore diameter: 4.6 µm), which consists of cotton linters fibers.

1.2 Instruments and characterization methods

A **Climate room** with a constant room temperature of 23 °C and 50 % rel. humidity was used to equilibrate all paper samples for at least 24 h prior to further use.

Grey values of fluorescence micrographs were determined by the software *ImageJ*.

NMR spectra of the bulk polymers were recorded by using a Bruker DRX300 (300 MHz) spectrometer at 25 °C and analysed by MestreNova (MestreLab Research S.L.). ^{1}H spectra are reported in δ units, parts per million (ppm) downfield from TMS (tetramethyl silane) and are calibrated by reference to residual chloroform (7.26 ppm) in the deuterated solvent.[S1]

Infrared spectra were recorded by using a PerkinElmer Spectrum One FT-IR spectrometer. The device was equipped with a single reflection UATR unit (PerkinElmer). Each individual spectrum was obtained by accumulation of 10 scans with a resolution of 1 cm^{-1} or 4 cm^{-1}.

Fluorescence microscopy was performed by an Olympus BX61 upright microscope providing a mercury arc lamp. Fluorescence micrographs were obtained by an Olympus XM10 camera using appropriate excitation and emission filters. Data were processed using Olympus AnalySIS software and image analysis was performed using ImageJ.

Photographs were captured with a Canon PowerShot SX220 HS (Canon Inc., Japan). Photographs, which were captured under UV light to visualize the polymer pattern on paper, were illuminated with an UV hand lamp (365 nm).

Photolithography was performed by using a 1000W Oriel flood exposure source (Newport, USA) equipped with an I-line filter at 365 nm. For illumination in defined regions lithography masks of emulsion film foil (printed with a resolution of 25,000 dpi; bvm maskshop, Germany) were used (for detailed shape of masks, see Supplementary Figure 1).

a) Spot pattern:

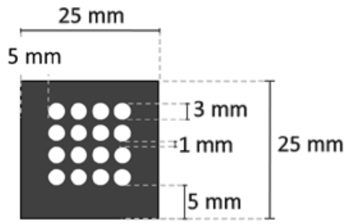

b) Y-shaped channels:

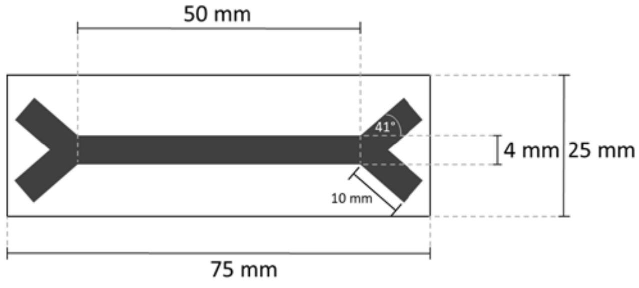

c) In-channel patches

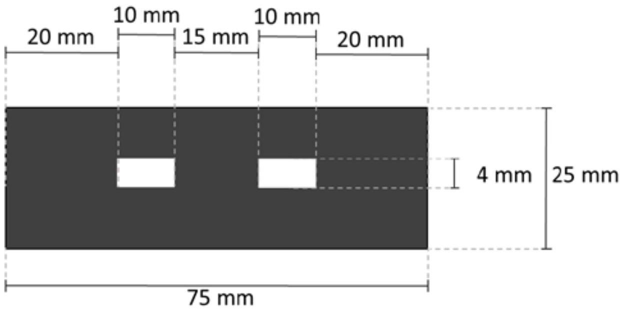

Supplementary Figure 1. Dimensions of photolithography masks: a) Spot pattern, b) Y-shaped channels, c) In-channel patches.[S2,S3,S4]

UV/Vis spectra were measured by using a VARIAN CARY 50 spectrophotometer (Varian Inc., USA).

2 Synthesis and production methods

2.1 Synthesis and characterization of compounds

Synthesis of aminoethyl rhodamine B (RhB-NH_2)

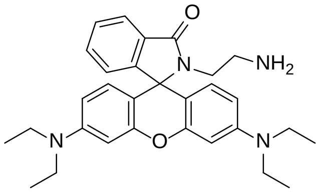

A solution of Rhodamine B base (1.78 g, 3.9 mmol) in ethanol (150 ml) is treated with ethylene diamine (3.05 g, 50 mmol) under nitrogen atmosphere and stirred at reflux temperature for 14 h. Subsequently, the solvent is evaporated under reduced pressure and the residue is dissolved in hydrochloric acid (1 M, 150 ml). Aqueous NaOH (1 M) is added carefully until the crude product preticipates as a pink solid. The crude product is filtered off, washed with water (3 × 50 ml) and vacuo to yield the desired compound as a pink solid (1.59 g, 79%). ^{1}H NMR ($CDCl_3$, δ in ppm): 1.16 (t, 12H), 2.94 (t, 2H), 3.33 (q, 10 H), 6.2-6.5 (m, 6H), 7.1 (s, 1H), 7.45 (t, 2H), 7.85 (d, 1H).

The analytical data are according to the data from the literature.[S4]

Synthesis of *N*-methacryloyl-β-alanine 1

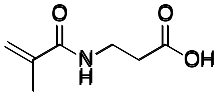

1

To an aqueous solution of β-alanine (1 M; 8.91 g in 100 ml of water) sodium bicarbonate (18.51 g, 220 mmol) and dioxane (50 ml) were added. Then the resulting mixture was cooled to 0 °C and a solution of methacryloyl chloride (10.3 ml, 105 mmol) in dioxane (50 ml) was added dropwise under nitrogen atmosphere. The reaction mixture was stirred at 22 °C for 15 h, followed by the addition of 100 ml water and subsequent washing with ethyl acetate (2 × 100 ml). The aqueous layer was acidified to pH 2 with 6 M aq.HCl at 0 °C and extracted with ethyl acetate (5 × 100 ml). After drying with $MgSO_4$, the solvent was removed under reduced pressure. For purification, the crude product was dissolved in ethyl acetate, precipitated from *n*-hexane at −21 °C for 14 h and dried *in vacuo* to obtain 6.61 g (42%) of the desired product **1**. ^{1}H-NMR ($CDCl_3$, δ in ppm): 1.95 (s, 3H), 2.65 (t, 2H), (q, 2H), 5.35 (s, 1H), 5.72 (s, 1H), 6.57 (broad s, 1H), 7.62 (broad s, 1H). The analytical data are consistent with the literature.[S5]

Synthesis of *N*-methacryloyl-β-alanine succinimide ester (MAC_2AE)

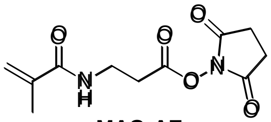

MAC_2AE

To a solution of *N*-methacryloyl-β-alanine **1** (2.0 g, 12.8 mmol) and *N*-hydroxysuccinimide (1.8 g, 12.8 mmol) in 25 ml of methylene chloride dicyclohexylcarbodiimide (DCC, 2.63 g, 12.75 mmol) was added at 0°C under nitrogen atmosphere. The mixture was stirred for 2 h at 0 °C and then kept at −21 °C for 16 h. The precipitate was removed via filtration and the residue washed with cold methylene chloride (0 °C, 5 × 50 ml). The solvent was evaporated and the crude product dried *in vacuo* and recrystallized from 2-propanol to yield the 2.03 g (63%) of the desired compound **MAC_2AE**. ^{1}H NMR ($CDCl_3$), δ (in ppm): 1.96 (s, 3H), 2.80-2.91 (m, 6H), 3.71-3.77 (q, 2H), 5.36 (s, 1H), 5.74 (s, 1H), 6.57 (broad s, 1H). The analytical data are consistent with the literature.[S5]

2.2 Synthesis of polymers

Synthesis of photo-crosslinkable polymer P(DMAA-*co*-MAC$_2$AE-*co*-MABP)

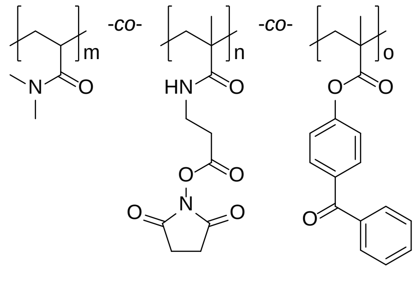

P(DMAA-*co*-MAC$_2$AE-*co*-MABP)

DMAA (1.74 g, 17.5 mmol) was added to a solution of *N*-methacryloyl-β-alanine succinimide ester (MAC$_2$AE, 0.51 g, 2 mmol) and MABP (0.133 g, 0.5 mmol) in DMF (20 ml) under nitrogen atmosphere. The mixture was treated with AIBN (2,2'-azobis(2-methylpropionitrile)), (9.5 mg, 0.06 mmol) and degassed by freeze-pumping (4 cycles). The mixture was stirred at 60 °C for 16 h; then the polymer was precipitated in diethyl ether (300 ml) and purified via reprecipitation with chloroform (20 ml) and diethyl ether (300 ml). The desired compound **Nr** was obtained as a colourless solid (1.34 g, 56%), Mn=26.000 g mol^{-1}; ^{1}H-NMR analysis gave a molar content of 11.5 mol% of MAC$_2$AE monomer and 2.3 mol% of photoreactive MABP.

The analytical data are according to the data from the literature.[S4]

Synthesis of P(S-*co*-MABP-*co*-PyMA)

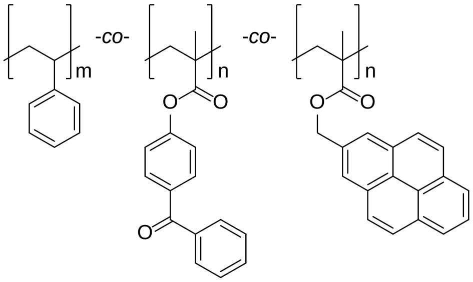

4-methacryloyloxybenzophenone (266.6 mg, 1.0 mmol) and 1-pyrenemethyl methacrylate (60.06 mg, 0.2 mmol) were added to styrene (3.96 g, 38.02 mmol) in a Schlenk flask under nitrogen atmosphere. The initiator AIBN (19.1 mg, 0.12 mmol) was added and the solution was degassed by four freeze-pump-thaw cycles. The flask was placed in a thermostated bath at 60 °C for 6 h for polymerization. After this time the solution was diluted with 15 ml THF and the polymer was precipitated in 300 ml methanol and dried in vacuum. The polymer was purified by repeated dissolution in THF and precipitation in methanol solution. The polymer was obtained as a slightly yellow solid (0.86 g, 20%), Mn = 82.000 g/mol. ^{1}H NMR analysis yielded a molar composition of about 4.5 mol% of the photo-reactive benzophenone group and about 3.5 mol% of the fluorescent monomer in the copolymer.

The analytical data are according to the data from the literature.[S4]

Synthesis of P(DMAA-*co*-MABP)

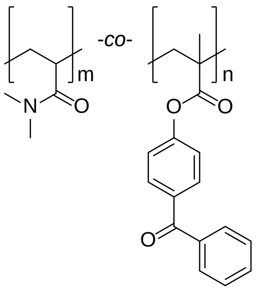

To a solution of MABP (226 mg, 1 mmol) in dioxane (50 ml) DMAA (3.86 g, 39 mmol) is added under nitrogen atmosphere. The solution is treated with AIBN (20 mg, 0.12 mmol), degassed by four freeze-pump-thaw cycles and stirred at 60 °C for 16 h. After this time, the crude product is precipitated in diethyl ether (650 ml), dissolved in acetone (30 ml) and precipitated in diethyl ether (300 ml). The polymer is purified by reprecipitation of THF (47 ml) and diethyl ether (500 ml) to yield the desired polymer as a white solid (3.0 g, 73%). M_n = 36.000 g mol^{-1}; ^{1}H NMR analysis gave a molar composition of 2.6 mol% of the photo-reactive benzophenone monomer.

The analytical data are according to the data from the literature.[S4]

Synthesis of P(DMAA-*co*-MAC$_2$AE-*co*-MABP-*co*-PyMA)

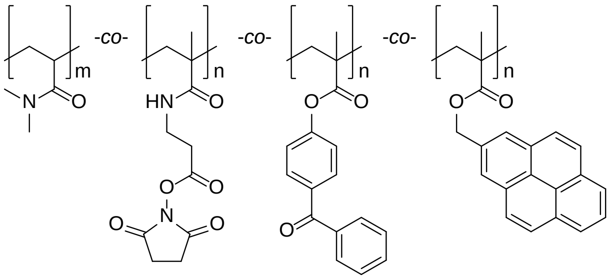

DMAA (1.72 g, 17.4 mmol) is added to a solution of MAC$_2$AE (510 mg, 2 mmol), MABP (133 mg, 0.5 mmol) and 1-pyrene methylacrylate (PyMa, 37 mg, 0.1 mmol) in DMF (20 ml) under nitrogen atmosphere. AIBN (9.7 mg, 0.06 mmol) is added and the solution is degassed by four freeze-pump-thaw cyles. Subsequently, the reaction mixture was heated to 60 °C for 16 h. After this time, the polymer is precipitated in diethylether (300 ml). The crude product was purified via reprecipitation with chloroform (20 ml) and diethylether (300 ml) to obtain the desired terpolymer as a pale yellow solid (yield: 1.4 g, 59%). ^{1}H NMR analysis gave a molar composition of 20 mol% of MAC$_2$AE, 2.3 mol% of MABP and 0.6 mol% of PyMA monomers in the polymer.

The analytical data are according to the data from the literature.[S4]

2.3 Covalent immobilization of polymers onto paper

General procedure for light-induced immobilization of MABP-containing polymers on paper

For covalent attachment of polymers to paper fibers, sheets were cut into the desired size and shape. Hydrophilic polymers (i.e. DMAA-based copolymers) were dissolved in $CHCl_3$ at a concentration of 30 mg ml^{-1}, whereas hydrophobic polymers were dissolved in THF at the same concentration (30 mg ml^{-1}). Subsequently, the paper substrates are coated with the polymer solution by dip-coating (submerging for 20 s) and air dried for 30 minutes. Next, the materials were placed inside a UV-exposure chamber and illuminated with UV light (wavelength λ = 365 nm, energy dose E = 16 J cm^{-2}) to induce crosslinking of the polymers. In order to generate well-defined polymer patterns on the paper surface, lithography masks were placed on top of the material during the illumination step. Finally, unbound molecules (polymers) were removed by Soxhlet extraction with $CHCl_3$ or THF for 2.5 h. Before further use, all samples were air dried and equilibrated for 24 h in a climate room.

Preparation of Y-shaped channels with P(S-*co*-MABP-*co*-PyMA) barrier

Paper (Roth Rotilabo® 15A) was cut into sheets of 7.5 × 2.5 cm, dip coated with P(S-*co*-MABP-*co*-PyMA) and subsequently photo-crosslinked according to the general procedure decribed above. During the illumination step, a photo-lithography mask with Y-shape was used to obtain a polymer functionalization in the desired pattern (see Supplementary Figure 1).

Generation of polymer patches of P(DMAA-*co*-MAC$_2$AE-*co*-MABP-*co*-PyMA)

The above described Y-channels were dip-coated with P(DMAA-*co*-MAC$_2$AE-*co*-MABP-*co*-PyMA) and subsequently photo-crosslinked according to the general procedure described above. During the illumination step, a photo-lithography mask with two rectangles was used to obtain a polymer functionalization in the desired pattern (see Supplementary Figure 1).

2.4 Preparation of the glucose sensor

The production of the glucose model sensor is schematically illustrated in Supplementary Figure 2. First, a microfluidic Y-channel was prepared via light-induced immobilization of P(S-*co*-MABP-*co*-PyMA) according to the procedure described above (section 2.3) (the shape of all lithography masks is described above in this document (section 1.2)) as well as in recent publications.[S2,S3,S4] In the next step, within this channel two polymerpatches of P(DMAA-*co*-MAC$_2$AE-*co*-MABP-*co*-PyMA) were attached in the same way (see section 2.3 and [S4])

Subsequently, one of the patches was treated with a solution of GOx-NH$_2$ in phosphate buffer (4 µl, approx. 0.8 units) and the other patch was treated with a solution of POx-NH$_2$ in phosphate buffer (4 µl, approx. 0.04 units). After a reaction time of 24 h at 5 °C, unbound molecules were removed via extensive rinsing of the material with water (5×100 ml) and shaking the material in a solution of NaCl (3 M, 100 ml) for 6 h at 22 °C. After rinsing again with water (100 ml), the paper sheet was air dried and a solution of Amplex Red in anhydrous DMSO (2.5 µl, 0.01 mol l^{-1}) was dropped onto the POx-functionalized patch in the paper channel.

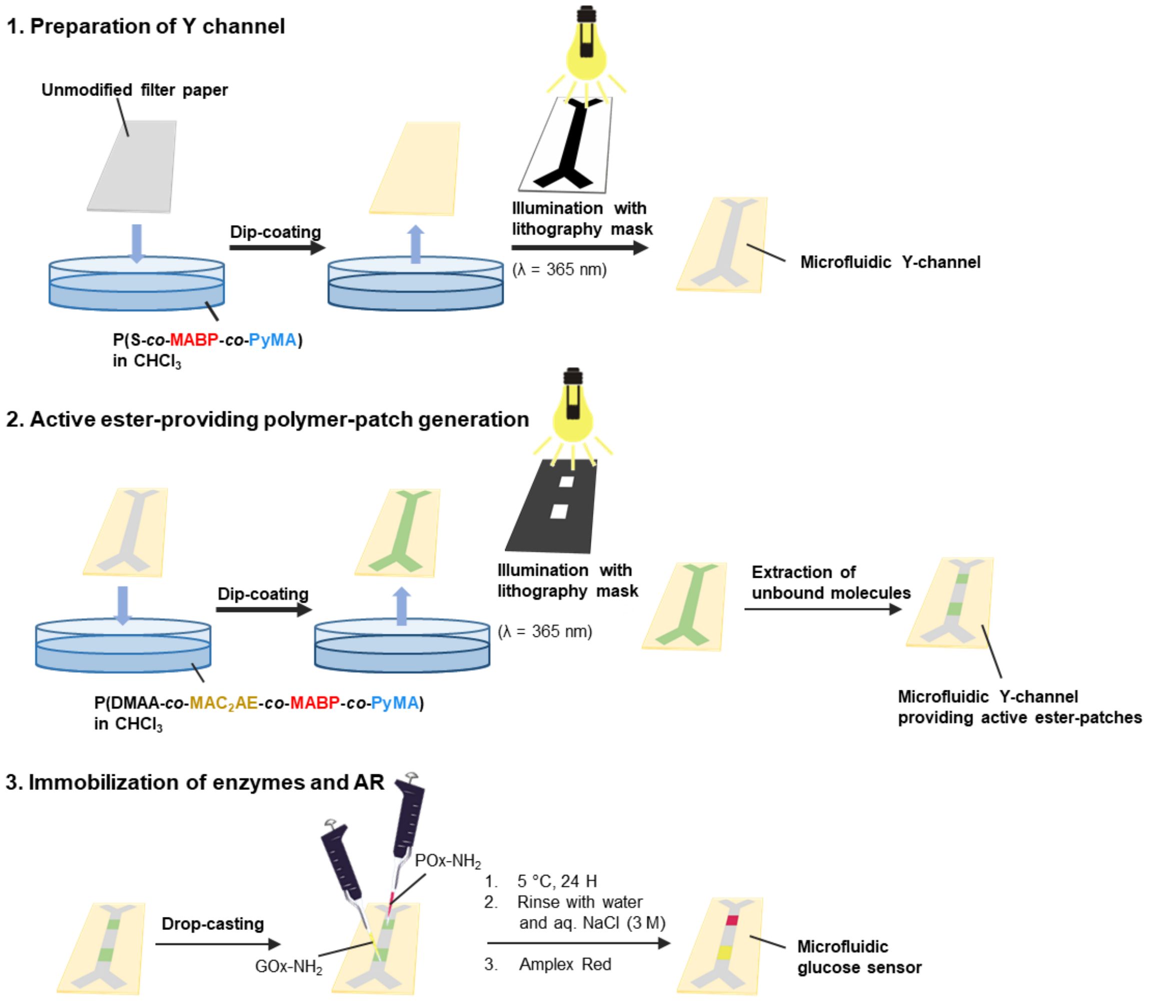

Supplementary Figure 2. Preparation steps of the glucose sensor: 1. Preparation of the Y-channel, 2. Active ester-providing polymer-patch generation, 3. Immobilization of POx, GOx and AR.

3 Reversible switching of RhB-modified paper

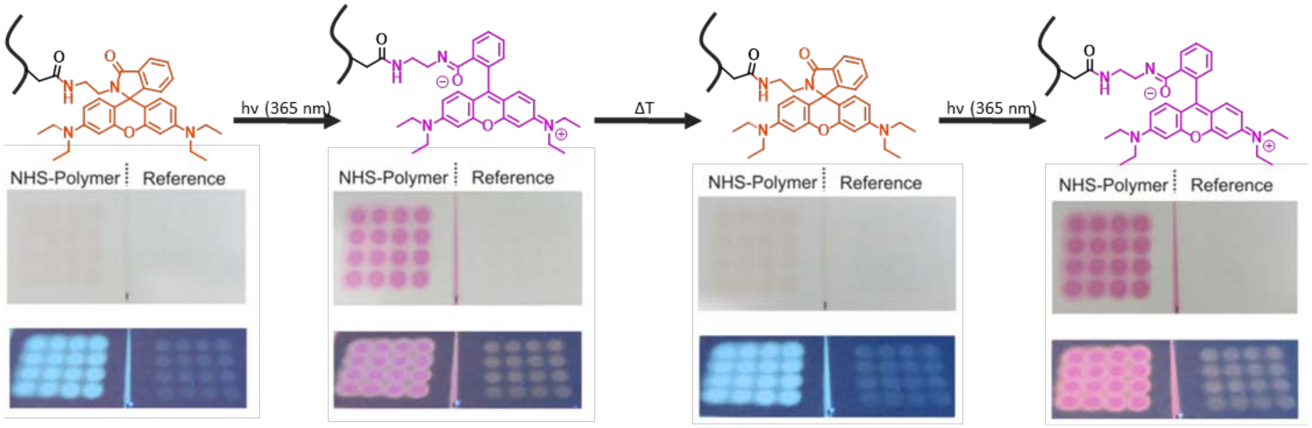

Supplementary Figure 3. Photographs showing reversible switching of RhB-modified paper under visible light and under UV light.

4 References

[S1] Fulmer, G. R., Miller, A. J. M., Sherden, N. H., Gottlieb, H. E., Nudelman, A., Stoltz, B. M., Bercaw, J. E., Goldberg, K. I. (2010). NMR Chemical Shifts of Trace Impurities: Common Laboratory Solvents, Organics, and Gases in Deuterated Solvents Relevant to the Organometallic Chemist. *Organometallics 29*, 2176-2179.

[S2] Böhm, A., Gattermayer, M., Carstens, F., Schabel, S., and Biesalski, M. (2013a). "Designing Microfabricated Paper Devices through Tailored Polymer Attachment". In Proceedings of the 15th FRC Symposium, Advances in Pulp and Paper Research, ed. S. j. I'Anson (FRC Cambridge, Cambridge (UK))

[S3] Wendenburg, S., Nachbar, M.-L., and Biesalski, M. (2017). Tailoring the Retention of Charged Model Compounds in Polymer Functionalized Paper-Based Microfluidic Devices. *Macromol. Chem. Phys.* 218, 1600408. doi: 10.1002/macp.201600408

[S4] Böhm, A. (2014) Grafting Photo-reactive Copolymers to Cellulose Fibers for the Design of Microfluidic Papers. [Dissertation]. [Darmstadt (Germany)]: Technische Universität Darmstadt.

[S5] Murata, H., Prucker, O., Rühe, J. (2007). Synthesis of Functionalized Polymer Monolayers from Active Ester Brushes. *Macromolecules 40*, 5497-5503.
